# Label-Free Comparative Proteomic Analysis Combined with Laser-Capture Microdissection Suggests Important Roles of Stress Responses in the Black Layer of Maize Kernels

**DOI:** 10.3390/ijms21041369

**Published:** 2020-02-18

**Authors:** Quanquan Chen, Ran Huang, Zhenxiang Xu, Yaxin Zhang, Li Li, Junjie Fu, Guoying Wang, Jianhua Wang, Xuemei Du, Riliang Gu

**Affiliations:** 1 Center for Seed Science and Technology, Beijing Innovation Center for Seed Technology (MOA), Key Laboratory of Crop Heterosis Utilization (MOE), College of Agronomy and Biotechnology, China Agricultural University, Beijing 100193, China; znchenquanquan@163.com (Q.C.); huangran086@126.com (R.H.); dlxuzhenxiang@163.com (Z.X.); zhangyaxin1994@126.com (Y.Z.); lili2016@cau.edu.cn (L.L.); wangjh63@cau.edu.cn (J.W.); 2 Institute of Crop Sciences, Chinese Academy of Agricultural Sciences, Beijing 100081, China; fujunjie@caas.cn (J.F.); wangguoying@caas.cn (G.W.)

**Keywords:** maize, black layer, seed development, proteomic analysis, stress response, MALDI-TOF MS

## Abstract

The black layer (BL) is traditionally used as an indicator for kernel harvesting in maize, as it turns visibly dark when the kernel reaches physiological maturity. However, the molecular roles of BL in kernel development have not been fully elucidated. In this work, microscopy images showed that BL began to appear at a growth stage earlier than 10 days after pollination (DAP), and its color gradually deepened to become dark as the development period progressed. Scanning electron microscopy observations revealed that BL is a tissue structure composed of several layers of cells that are gradually squeezed and compressed during kernel development. Laser-capture microdissection (LCM) was used to sample BL and its neighboring inner tissue, basal endosperm transfer layer (BETL), and outer tissue, inner epidermis (IEP), from 20 DAP of kernels. Matrix-assisted laser desorption/ionization time-of-flight mass spectrometry profiling (MALDI-TOF MS profiling) detected 41, 104, and 120 proteins from LCM-sampled BL, BETL, and IEP, respectively. Gene ontology (GO) analysis indicated that the 41 BL proteins were primarily involved in the response to stress and stimuli. Kyoto Encyclopedia of Genes and Genomes (KEGG) pathway analysis found that the BL proteins were enriched in several defense pathways, such as the ascorbate and aldarate metabolic pathways. Among the 41 BL proteins, six were BL-specific proteins that were only detected from BL. Annotations of five BL-specific proteins were related to stress responses. During kernel development, transcriptional expression of most BL proteins showed an increase, followed by a decrease, and reached a maximum zero to 20 DAP. These results suggest a role for BL in stress responses for protecting filial tissue against threats from maternal sides, which helps to elucidate the biological functions of BL.

## 1. Introduction

Maize is one of the most important grain crops with a total production of more than one billion tons, accounting for ~30% of the world’s food supply (https://www.statista.com). Harvesting in proper time is a key factor to obtain high-quality kernels and avoid yield loss in maize production [1,2,3]. Black layer (BL) development has been widely used as an indicator of kernel maturity in agronomy, as it turns visibly dark when the kernel reaches physiological maturity (maximum kernel weight) in maize. The physiological relationship between BL formation and kernel maturity has been intensively investigated in recent decades [4,5,6,7,8,9], but the developmental processes, as well as the molecular roles, of BL have not been fully elucidated.

BL comprises several layers of cells located between the filial endosperm and the maternally derived pericarp and belongs to a major bridge structure, known as the placento-chalazal (P-C) layer. BL is derived from the inner integument and the integumental P-C (iP-C), which is located immediately below another P-C layer, nucellar P-C (nP-C) which is immediately subtended from the endosperm and is derived from the nucellus epidermis [10]. At the early kernel development stage (before 10 days after pollination, DAP), programmed cell death process (PCD)-based cell death was observed in BL [11], which leads to cell degradation into a narrow band of crushed cells with accumulated brown pigment and subsequently leads to the emergence of visible BL [11,12]. These visible BLs are generally used as kernel maturity indicators by agronomists and farmers.

Cells above the P-C layer include several layers of endosperm cells called the basal endosperm transfer layer (BETL), and cells below the P-C layer are the inner epidermal cells of the pedicel (IEP). The P-C layer, together with the BETL and pedicel, function as the transfer of photoassimilates and nutrients from the mother plant to the filial caryopsis. In maize, phloem tissue is transported in the pedicel. However, this tissue does not extend beyond the pedicel. Thus, a different transport system is expected to exist in the P-C layer and BETL after the phloem termini in the pedicel [11,12,13]. Nevertheless, whether BL is involved in this critical role of post-phloem transport has not been determined.

Although described in various plant species, a P-C region is known to exhibit a high level of anatomical variability in its structural adaptations [14]. P-C layers are believed to possess the best developed structure in tropical crops, such as maize and sorghum, which show assimilated transport from only the base of the caryopsis [11,15]. Thus, investigating BL development and predicting its biological roles are particularly interesting in maize.

To date, the rapid development of proteomic technologies provides a notable opportunity for plant proteomic profiling [16]. Proteomics studies in maize whole kernel development have been intensively conducted at different scales of genotype, growth stage, and treatment [17,18,19,20,21,22,23]. Proteomics has also been performed in specific kernel tissue of BETL and cellular component of protein body to investigate their special function and developmental process [24,25]. However, no attempt has been made to attach to BL tissue. In addition, most of the abovementioned proteomics analyses were generally conducted by the two-dimensional (2D) gel-based mass spectrometry method. Recently, matrix-assisted laser desorption/ionization time-of-flight mass spectrometry profiling (MALDI-TOF MS profiling) is an emerging approach based on rapid and high-throughput screening of ions from molecules directly detected in biological samples [26], which has been applied in plant protein identification [27].

In this work, cells of BL together with its neighboring tissues, BETL and IEP, were obtained by laser-capture microdissection, and their accumulated proteins were identified by the MALDI-TOF MS method. The networks and putative functions of BL-expressed proteins were further discussed. These results could help to elucidate the mechanisms of BL formation and the biological roles of this special structure.

## 2. Results

### 2.1. Observation of Black Layer Development in Maize Kernels

**Figure 1:**
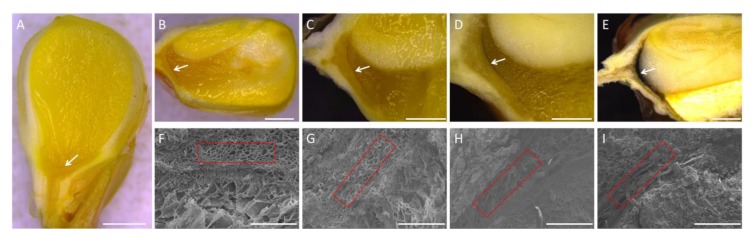
Observation of the black layer in maize kernels at different developmental stages. (**A**–**E**) Longitudinal section of maize kernels 10, 20, 30, 40, and 50 days after pollination (DAP). The arrowhead indicates the position of the black layer. Bar = 2 mm; (**F-I**) Scanning electron microscopy analysis of the black layer of maize kernels 20, 30, 40, and 50 DAP. Regions surrounded by red boxes indicate the structure of the black layer. Bar = 200 μm.

The kernel development was investigated 10, 20, 30, 40, and 50 DAP (Figure 1). The embryo structure was unobservable 10 DAP, while its structure outline could be observed 20 DAP. This result is consistent with previous findings that embryo structure is differentiated from the endosperm 10 to 20 DAP [28,29].

Ten DAP, a band with a different color from the surrounding area of the endosperm could be observed at the base of the kernel, which was likely to form BL during the latter development (Figure 1A). From 10 to 40 DAP, the color of this band gradually changed to black (Figure 1A–D), while from 40 to 50 DAP, the color changed dramatically, and a dark black band emerged 50 DAP (Figure 1D, E). These results suggested that BL development could start earlier than embryo differentiation at a stage before 10 DAP and progress to seed maturity 50 DAP.

From the electron-microscope analysis, it was observed that BL was composed of several layers of cells. This structure was gradually compressed and finally became a small band along with kernel development from 20 DAP to 50 DAP (Figure 1F–I). This result raised the possibility that the color change of BL from light to dark was partially due to a concentration effect of brown pigment cells.

### 2.2. Isolation of Black Layer Cells

**Figure 2:**
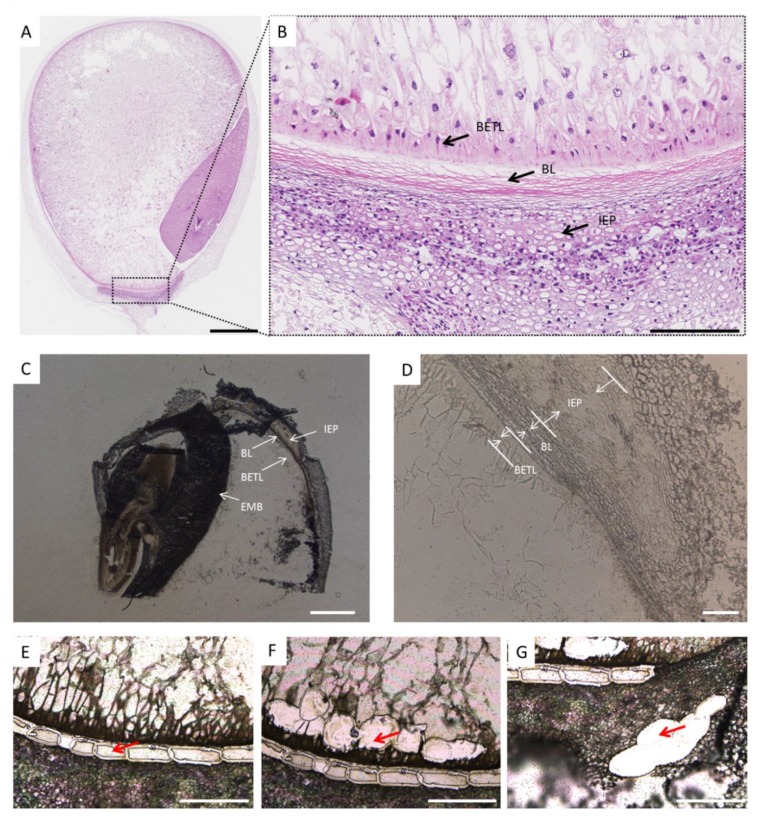
Isolation of the black layer (BL), inner epidermis of the pedicel (IEP), and basal endosperm transfer layer (BETL) from maize kernels 20 DAP by laser-capture microdissection. (**A**) Longitudinal paraffin sections of maize kernels 20 DAP. Bar = 1.25 mm; (**B**) High-magnification longitudinal sections of maize kernels 20 DAP. Arrows indicate the position of BL, IEP, and BETL. Bar = 200 μm; (**C**) and (**D**) Tissues from 8 μm thick cryosections before LCM cutting, showing the IEP, BL, BETL, and EMB. EMB, embryo. (**C**) Bar = 1000 μm. (**D**) Bar = 100 μm; (**E**–**G**) Tissues from 8 μm thick cryosections after LCM cutting. The red arrow indicates the successfully cut tissues of BL (**E**), BETL (**F**), and IEP (**G**). Bar = 200 μm.

Since BL begins to form as early as 10 DAP, 20 DAP kernels were selected for proteomic analysis because the proteins involved in BL development are expected to accumulate at this stage. Furthermore, the longitudinal section of the whole kernel indicated that BL, BETL, and IEP could be clearly distinguished at 20 DAP (Figure 2A–D). Thus, BL, as well as its neighboring tissues BETL and IEP, were successfully collected after subjecting kernel section and Laser-Capture Microdissection (LCE) under high microscope resolution conditions (Figure 2E–G).

### 2.3. Proteomic Analysis of the Black Layer Cells

**Figure 3:**
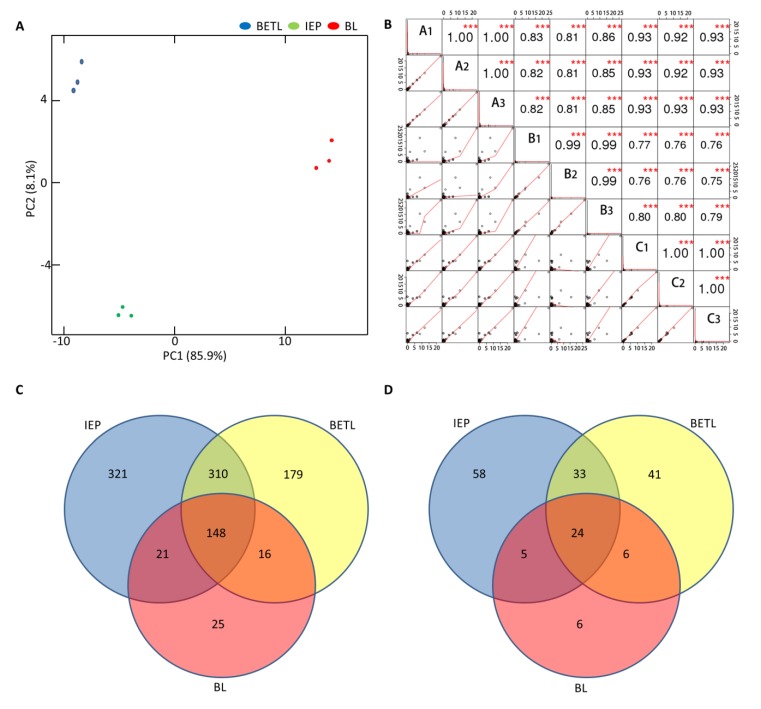
Proteomic analysis of peptides and protein identified from the black layer (BL), inner epidermis of pedicel (IEP), and basal endosperm transfer layer (BETL), of maize kernel 20 DAP. (**A**) Principle component analysis (PCA) of the identified peptides from the three replicated samples of IEP, BL, and BETL; (**B**) Coefficiency correlation analysis of the identified peptides from the three replicated samples of IEP, BL, and BETL. A (1, 2, 3), B (1, 2, 3), and C (1, 2, 3) represent the three replicates of IEP, BL, and BETL, respectively. (**C**) Venn diagram of the identified peptides from IEP, BL, and BETL. (**D**) Venn diagram of the identified proteins from IEP, BL, and BETL.

Proteins were identified by quadrupole time-of-flight mass spectrometry. The MS data were searched against the UniProt Plant protein database containing 87,406 sequences (http://www.uniprot.org) and the NCBI maize database containing 280,238 sequences (http://www.ncbi.nlm.nih.gov), which resulted in 210, 800, and 653 peptides from BL, IEP, and BETL, respectively (Table S1). These peptides represented 1020 non-redundant peptides (Figure 3C). Using these peptides, PCA analysis showed that the three biological replicates for each tissue were closely related and grouped together (Figure 3A). PC1 accounted for 85.9% of the total component. From this component, the BETL and IEP groups showed a closed relationship, but they were located far from the BL group. Meanwhile, the correlation analysis showed notably high positive correlations among the replicates within a tissue (*R*^2^ = 0.99 to 1.00) and high positive correlations across tissues (*R*^2^ = 0.75 to 0.93). Furthermore, correlations between BL and either BETL (*R*^2^ = 0.75 to 0.77) or IEP (*R*^2^ = 0.81 to 0.86) were lower than those between BETL and IEP (*R*^2^ = 0.92 to 0.93) (Figure 3B). The PCA and correlation analyses indicated that the protein accumulation in BL was significantly different from those in the other two tissues.

**Figure 4:**
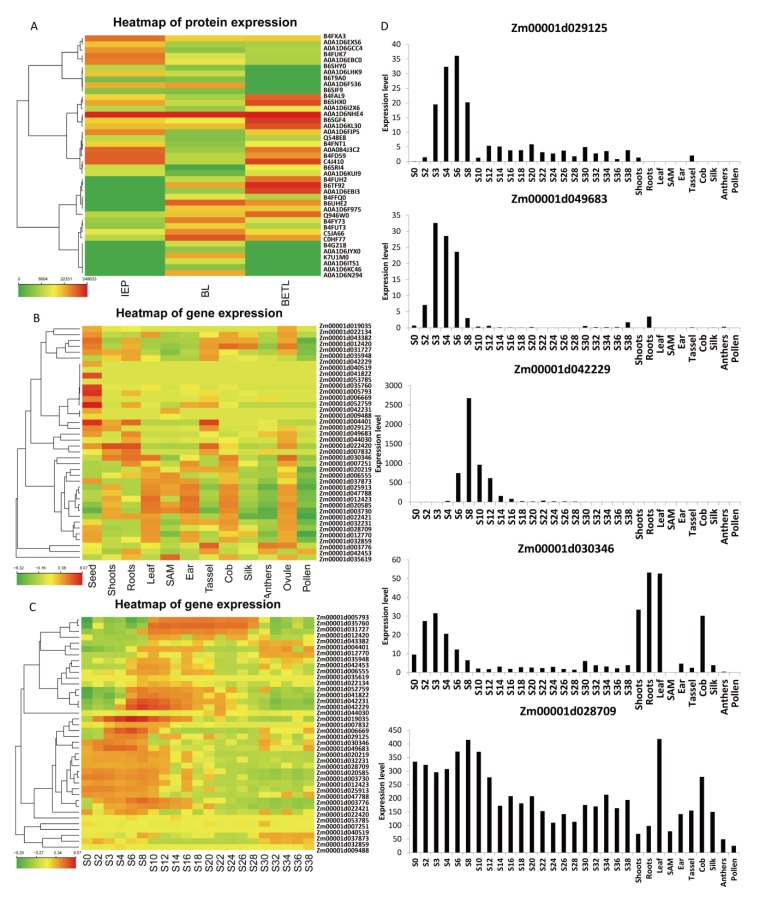
Expression patterns of proteins and genes identified from the black layer (BL). (**A**) Heatmap cluster of proteins identified from BL. Expression pattern analysis of BL genes in maize tissues (**B**) and during seed development (**C**). (**D**) Expression of the 5 BL-specific expression genes in maize tissues and during seed development. The normalized values after log_2_ transformation of reads per kilobase per million (RPKM) were used for (**B**) and (**C**). S represents seed and number represents days after pollination. Expression data were extracted from Chen et al. (2014) [30].

The 1020 peptides represented 173 nonredundant protein entrances (Table S2). Among these proteins, 41 were recognized in BL, which was considerably lower than the number detected in IEP (120) and BETL (104) (Figure 3D). Of the 41 BL proteins, six uniquely accumulated in BL, and 24 constitutively accumulated in all three tissues from a heatmap analysis of their protein accumulation (Figure 4A). The lowest total protein number and tissue-specific protein number detected from BL suggested a lower metabolic activity of this tissue as compared with its neighboring tissues.

### 2.4. Gene ontology Annotation Function and Kyoto Encyclopedia of Genes and Genomes Analysis of Proteins Identified from BL

**Figure 5:**
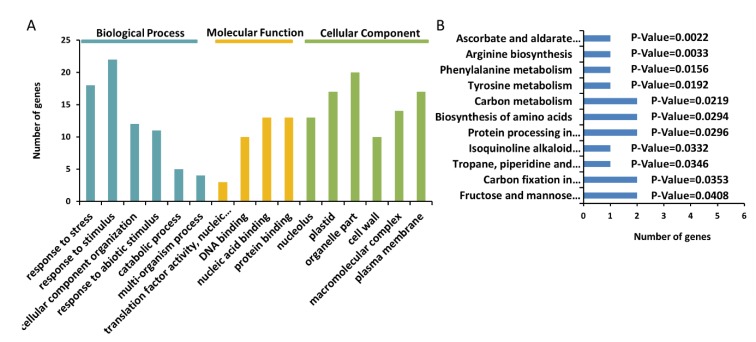
Gene ontology (GO) annotation function (**A**) and Kyoto Encyclopedia of Genes and Genomes (KEGG) (**B**) analyses of the identified proteins from the black layer.

Gene ontology (GO) (http://systemsbiology.cau.edu.cn/agriGOv2/) analysis of the 41 BL accumulated proteins revealed six GO terms for cellular components and biological processes and four for molecular functions (Figure 5A and Table S3). In the biological process category, the largest subcategory was response to stimulus followed by response to stress. For the cellular component category, the subcategories were significantly enriched at the organelle part, plastid, nucleolus, plasma membrane, macromolecular complex, and cell wall. For molecular function, the top four subcategories with highly significant p-values were nucleic acid binding, protein binding, DNA binding, and translation factor activity.

Kyoto Encyclopedia of Genes and Genomes (KEGG) pathway analysis was further performed for the 41 BL proteins, which mapped to 11 KEGG pathways (Figure 5B). Five pathways were significantly enriched in different metabolic processes, including a pathway in the ascorbate and aldarate metabolic pathways that played protective roles in plants, particularly under stress conditions.

Except for one unknown protein, five out of the six BL-specific proteins were cytosolic ascorbate peroxidase protein, pathogenesis-related protein, salt stress-induced protein, desiccation-related protein, and small heat shock protein. All five of these proteins have been reported to be involved in stress responses.

### 2.5. Gene Expression of BL Accumulated Proteins

According to a heatmap analysis for the 41 BL proteins, the accumulation of 14 proteins showed higher levels in BL than in IEP and BETL. Of these 14 proteins, six were specifically expressed in BL (Figure 4A). From a published dataset [30], 38 out of the 41 BL genes had expression records. Interestingly, most of the BL genes showed high expression levels in seeds as compared with other plant organs (Figure 4B and Table S4). In addition, 33 genes showed an increasing and, then, a decreasing expression pattern along with the seed development process, with 17 genes showing the highest level at the early seed development stage (zero to 10 DAP) and 16 at the middle stage (six to 22 DAP) (Figure 4C). As gene transcription always started slightly earlier than protein accumulation, the high transcription levels of these genes from zero to 22 DAP were consistent with the results of protein profiling at 20 DAP.

Concerning approximately the five BL-specific genes, Zm00001d029125, Zm00001d049683, and Zm00001d042229 showed exclusive expression in seeds, particularly at seeds three to 12 DAP (Figure 4D). Zm00001d028709 and Zm00001d030346 showed constitutive expression in many plant organs, including seeds. Within seeds, Zm00001d028709 was expressed during the whole seed developmental process, while Zm00001d030346 showed high levels at the early developmental stage.

## 3. Discussion

As proteins are the critical executors that directly participate in life activities, the study of proteomics can make significant contributions to elucidating the mechanism of important biological processes. The identification of proteins in previous works was mainly performed by two-dimensional electrophoresis, which has been considered a method with low specificity, efficiency, and accuracy [31]. In recent years, liquid chromatography-mass spectrometry (LC-MS) technology has become the preferred method for spatial proteomics (the localizations of proteins and their dynamics at the subcellular level), which could show the quantitative state of a proteome. On the basis of the MS method, several new technologies have been developed for quantitative proteomics [32], including label-free iTRAQ, SILAC, MRM (MRMHR), and SWATH [33,34,35,36,37]. In this paper, the label-free iTRAQ method was used for MALDI-TOF MS profiling.

Maize kernel is a single-seeded fruit that consists of a large embryo and a triploid endosperm encased in a maternally originated pericarp. BL is located between the filial endosperm and the maternally derived pericarp and belongs to a major bridge structure called the P-C layer. Neighboring inside the BL is an endosperm tissue called BETL, and outside is a pericarp IEP. The proteome of whole maize seeds, embryos, and endosperm has been intensively investigated in recent decades [23,25,38,39,40,41,42,43,44]. Due to the difficulty of separating tissues from kernels, the proteome for some targeted seed parts has seldom been the focus, except for BETL, whose functions have been implied to mediate embryo–endosperm interactions and play a role in plant defense by this method [45,46,47,48] To date, no attempt has been focused on BL, and very little is known about its developmental processes, as well as the physiological and molecular roles of this tissue in kernel development. In this study, we obtained BL tissue by the LCE method and analyzed its protein accumulation by MALDI-TOF MS. BL was located between BETL and IEP from the observation of microscope image (Figure 1), but its protein accumulation was significantly different from its neighboring tissues IEP and BETL (Figure 3A,B). In addition, detectable protein from BL was considerably lower than those from the neighboring tissues (Figure 3C,D), suggesting a distinct behavior of lower metabolic activity for BL.

BETL has the functions of transporting nutrients from the maternal to the endosperm and embryo of the seed. As BL is located outside the BETL, we speculated that BL plays a connecting role between BETL and IEP. Compared to several transport proteins, e.g., ion channel protein and aminotransferase protein, identified from BETL, no transport-related proteins could be found from BL (Table S2). However, three genes were found to be related to cell death. Zm00001d043382 was found by GO functional annotation with an encoded protein involved in the senescence pathway (GO:0010149) [49]. Zm00001d037873 and Zm00001d012770 encode proteins with GTPase activity (GO:0003924), which has been reported to be associated with cell death [50,51]. This finding was consistent with those of previous publications, where PCD-based cell death was previously observed at a stage as early as 10 DAP in BL [11]. As cell death would lead BL cells to lose their cellular activity during the later seed development stage, the death of BL cells would block nutrient flow through this tissue. Thus, these results suggested that BL could have a special role, other than nutrient transport, in seed development.

GO and KEEG analyses indicated that the BL-accumulated proteins showed functions related to the stress response (Figure 5A,B). Of the six BL-specific proteins, Zm00001d028709 encodes the cytosolic ascorbate peroxidase Apx1 and Zm00001d049683 encodes a homolog of rat L-gulono-1,4-lactone (L-GulL) oxidase that is involved in the biosynthesis of L-ascorbic acid. Ascorbic acid reacts with H_2_O_2_ under the action of ascorbic peroxidase, thereby, eliminating the toxicity of H_2_O_2_ [52,53,54,55]. This process plays a significant role in the stress resistance of plants. Zm00001d030346 encodes a small heat shock protein 21 (Hsp21), which plays a crucial role in protecting plants against stress by re-establishing cellular homeostasis in the abiotic stress response, plant disease resistance, and oxidative stress [56,57,58,59]. As Apx1 and Hsp can be involved in the same metabolic network and play a protective role in plants [52,60], these results suggest that Zm00001d028709, Zm00001d049683, and Zm00001d030346 function in stress responses under a connected pathway within BL.

Zm00001d042229 was predicted to encode a PR (pathogenesis-related) protein, which possesses antimicrobial properties involved in the biotic and abiotic stress responses of plants. A protective role for the embryo surrounding the region of the maize endosperm was previously demonstrated by the characterization of *ZmESR-6*, a defensin gene specifically expressed in this region [48]. A similar protective role for the filial surrounding region of the maternal tissue could be verified after further functional characterization of the BL-specific accumulation gene Zm00001d042229.

Zm00001d029125 encodes the desiccation-related protein PCC13-62 precursor. Cellular desiccation regulates the maturation of seeds [61,62,63]. Thus, the desiccation of BL cells along with PCD-based cell death blocks the transport of nutrients between BETL and IEP tissues, which plays a role in protecting the endosperm and embryo of seeds. This possibility was supported by evidence from previous works. For example, the basal layer-type antifungal protein 2 (BAP2) protein accumulated in BL and exhibited broad-range activity against a range of filamentous fungi [64]. Similar cellular autolytic events have also been reported in the nucellar projection (a tissue similar to BL) in barley [65].

## 4. Materials and Methods

### 4.1. Plant Materials and Sample Collection

PH6WC, the maternal line of an elite hybrid XY335 [3], was grown in 2017 at Zhuozhou, Hebei Province (39.486°N 115.974°E). Ears were manually self-pollinated and harvested 10, 20, 30, 40, and 50 days after pollination (DAP). For each stage, the harvests were repeated three times serving as the three biological replicates. Seeds from the middle part of each cob were carefully sampled for further analysis. Seeds were cut longitudinally with a scalpel and observed under a photomicroscope (Leica S9 i; Leica, Wetzlar, Germany). Seeds were cut into small cubes (approximately 10 × 10 mm) by a dissecting scalpel for laser-capture microdissection (LCM) following a method described by Zhu et al. [66]. These cubes were immediately submerged in a precooled fixative solution (75% (*v*/*v*) ethanol and 25% (*v*/*v*) acetic acid) at a 1:10 volume ratio of cube ice for 15 min and transferred into a new fixing solution overnight with gentle rotation at 4 °C. Cubes were transferred into 10% *w*/*v* sucrose in phosphate buffered saline (PBS) buffer (137 mM NaCl, 8 mM Na_2_HPO4, 2.7 mM KCl, and 1.5 mM KH_2_PO4, pH = 7.3) containing protease inhibitor (1:1000 volume ratio dilution) (Sigma, St Louis, MO, USA), followed by infiltration under vacuum on ice for 15 min. Cubes were transferred to 20% *w*/*v* sucrose in the same PBS and protease inhibitor buffer for another 15 min of infiltration. Then, the cubes were washed with optimum cutting temperature (OCT) medium (Tissue-Tek, Sakura, Japan), transferred into Eppendorf tubes supplied with OCT medium, and frozen with liquid nitrogen. The frozen tissue cubes were placed on ice for immediate microsection or stored at −80 °C.

### 4.2. Light and Scanning Electron Microscopy

Kernels were collected 20 DAP and cut along the longitudinal axis for imaging under light microscopy. Tissues containing the embryo part were fixed for 3 days at room temperature in FAA solution (38% formaldehyde 5 mL/glacial acetic acid 5 mL/70% ethanol 90 mL). The material was embedded in paraffin by dehydration in an ethanol gradient series (70%, 80%, 95%, and 100% ethanol) and subsequently cut into 8 µm sections. The sections were stained with toluidine blue and observed using a Nikon Ti microscope (Nikon, Melville, NY, USA).

Kernels were collected 20, 30, 40, and 50 DAP as samples for scanning electron microscopy. Kernels were critically dried, and sputter coated with gold. Gold-coated samples were observed with a scanning electron microscope S-3400N (Hitachi, Tokyo, Japan).

### 4.3. Laser Capture Microdissection

The tissues were sectioned at 8 μm in a cryostat (CM3050S; Leica, Wetzlar, Germany) and mounted on an adhesive-coated slide at −25 °C, as described by Nakazono et al. [67]. The sections were immediately incubated in 70% (*v*/*v*) ethanol at −20 °C for 1 min and washed with precooled ddH_2_O for 30 s followed by dehydration steps of 1 min each in 95% ethanol and 100% ethanol and 2 min twice in xylene. The sections were air-dried and used intermediately for LCM. During the LCM process, BETL, BL, and IEP cells were isolated using the following parameters: 7.5 μm laser spot size, 50 mW laser power, and 550 to 650 μs laser pulse duration in the PixCell Ⅱ LCM system (Arcturus Bioscience, Carlsbad, CA, USA) [67].

### 4.4. Protein Extraction

The isolated tissues were transferred to 0.2 mL tubes containing 30 µL extraction solution (6 M urea, 50 mM dithiothreitol, 0.5 M Tris-HCl, pH 8.0, 1:1000 diluted protease inhibitor). Proteins were dissolved by shaking the tube and, then, incubated on ice for 15 min. For efficient protein extraction, the tissue sample was cracked by ultrasonication on ice under an ultrasonic parameter of 3 seconds 30 Watt power supply interrupted by 6 seconds 50 times. After centrifugation at 4 °C and 14,000 g for 40 min, the supernatant was transferred to a clean 2 mL tube for protein digestion, which was performed using the FASP procedure described by Wisniewski et al. [68]. DTT was added into the tube to a final concentration of 10 mM for incubation at 37 °C for 1 h. IAA was added to a final concentration of 30 mM for reaction at 37 °C for 30 min in the dark. Then, 50 mM NH_4_HCO_3_ was added to a final volume of 1 mL. Five micrograms of trypsin were added to this solution for protein digestion at 37 °C overnight. After adding 10% (*v*/*v*) formic acid to a final concentration of 1% and reacting for 5 min, the supernatant containing peptides was collected by centrifugation at 4 °C and 14,000 g for 10 min, which served for desalt filtration by C18 cartridges (Empore™ SPE Cartridges C18 standard density, bed I.D. 7 mm, volume 3 mL, Sigma). Then, the peptides were concentrated by vacuum and redissolved in 20 μL 0.1% (*v*/*v*) formic acid. The concentration of peptides was determined by UV spectrometry at 280 nm [69].

### 4.5. Proteomics Analysis

Proteins were identified in a quadrupole time-of-flight mass spectrometer (Agilent model 6500, Wilmington, DE, USA) following the user manual of MALDI-TOF MS. Using MaxQuant software 1.3.0.5, the MS data were searched against the UniProt Plant protein database containing 87,406 sequences (http://www.uniprot.org) and the NCBI maize database containing 280,238 sequences (http://www.ncbi.nlm.nih.gov). The search parameters were set as: ± 20 ppm for peptide mass tolerance, 0.1 Da fragment mass tolerance, and 2 maximum missed cleavages. The cutoff for the global false discovery rate (FDR) in peptide identification was 0.01. To reduce the probability of false identification, only peptides with significance scores at the 95% confidence interval were counted as identified peptides, and peptides repeatedly identified from at least two out of the three replicates were considered proteins that were expressed in the corresponding tissue. Intensity-based absolute quantification (iBAQ) in MaxQuant was performed to quantify peptide abundance for each replicate, with the cutoff values *p* = <0.05 and FDR = <0.05. The reproducibility of the triplicates for all tissues was analyzed using the iBAQ data by coefficiency correlation analysis (Pearson’s) at a significance level of *p* < 0.05 and principal component analysis (PCA). Then, the mean iBAQ data from the three replicates served as the abundant data for each peptide, which were used for identification of the corresponding protein under threshold of fold changes of >1.5 or < 0.67 at *p* value < 0.05.

Functional pathways were analyzed using gene ontology (GO) functional enrichment analysis (https://david.ncifcrf.gov/), where the GO categories included biological processes, molecular function, and subcellular locations. In addition, the significant biological process-associated proteins were selected to map to the pathways in KEGG (https://www.genome.jp/kegg/).

### 4.6. Expression Analysis of Candidate Genes

Raw datasets of RNA-Seq from different maize tissues were extracted from RNA-seq libraries and used for heatmap analysis of candidate genes [30]. The details about the data sources are described in Table S2. The raw reads were aligned to the B73 reference genome (RefGen_v2) using Tophat 2.0.6 (http://ccb.jhu.edu/software/tophat/index.shtml; Trapnell et al., 2009) with maximum intron length set to 30 kb, with default settings for other parameters. The number of uniquely mapped reads for each gene model in B73 was calculated by parsing the alignment output files from Tophat and, then, normalizing the resulting read counts by reads per kilobase per million (RPKM) to measure the gene expression level. Uniquely mapped reads were used to estimate the normalized transcription level, and the normalized value after log_2_ transformation of RPKM was used for Figure 4.

## 5. Conclusions

In this work, microscopy observation revealed that BL began to appear at a growth stage earlier than 10 DAP. Cells of BL, BETL, and IEP were successfully collected from 20 DAP kernels by LCM and used for protein profiling by the MALDI-TOF MS method. The BL-accumulated proteins were primarily enriched in functions of stress responses. By comparing BETL and IEP, six BL-specific proteins were identified, with five showing high gene expression at the early kernel development stage and homology to previously reported plant stress-responsive genes. Thus, the results of this study suggest a special role for BL in protective functions.

## Supplementary Materials

The following are available online at https://www.mdpi.com/1422-0067/21/4/1369/s1.

## Abbreviations

BL	black layer
DAP	days after pollination
BETL	basal endosperm transfer layer
IEP	inner epidermis cells of the pedicel
GO	Gene ontology
KEGG	Kyoto Encyclopedia of Genes and Genomes
P-C	placento-chalazal
iP-C	integumental P-C
nP-C	nucellar P-C
PCD	program cell death process
LCM	laser-capture microdissection
PBS	phosphate buffered saline
OCT	optimum cutting temperature
FDR	false discovery rate
iBAQ	Intensity-based absolute quantification
PCA	principal component analysis
L-GulL	L-gulono-1,4-lactone
Hsp21	heat shock protein21
BAP2	basal layer-type antifungal protein
MALDI-TOF MS	Matrix-assisted laser desorption/ionization time-of-flight mass spectrometry profiling
